# Resilience of swine nasal microbiota to influenza A virus challenge in a longitudinal study

**DOI:** 10.1186/s13567-023-01167-9

**Published:** 2023-05-02

**Authors:** Samantha J. Hau, Daniel W. Nielsen, Kathy T. Mou, David P. Alt, Steven Kellner, Susan L. Brockmeier

**Affiliations:** 1 USDA, ARS, National Animal Disease Center, Ames, IA USA; 2 ORAU/ORISE, Oak Ridge, TN USA

**Keywords:** Swine, microbiome, influenza A virus, porcine respiratory disease complex

## Abstract

Influenza A virus (IAV) is an important contributing pathogen of porcine respiratory disease complex (PRDC) infections. Evidence in humans has shown that IAV can disturb the nasal microbiota and increase host susceptibility to bacterial secondary infections. Few, small-scale studies have examined the impact of IAV infection on the swine nasal microbiota. To better understand the effects of IAV infection on the nasal microbiota and its potential indirect impacts on the respiratory health of the host, a larger, longitudinal study was undertaken to characterize the diversity and community composition of the nasal microbiota of pigs challenged with an H3N2 IAV. The microbiome of challenged pigs was compared with non-challenged animals over a 6-week period using 16S rRNA gene sequencing and analysis workflows to characterize the microbiota. Minimal changes to microbial diversity and community structure were seen between the IAV infected and control animals the first 10 days post-IAV infection. However, on days 14 and 21, the microbial populations were significantly different between the two groups. Compared to the control, there were several genera showing significant increases in abundance in the IAV group during acute infection, such as *Actinobacillus* and *Streptococcus*. The results here highlight areas for future investigation, including the implications of these changes post-infection on host susceptibility to secondary bacterial respiratory infections.

**Supplementary Information:**

The online version contains supplementary material available at 10.1186/s13567-023-01167-9.

## Introduction

Influenza A virus (IAV) is an orthomyxovirus that causes respiratory disease in humans and other animals, including pigs. In pigs, IAV contributes to porcine respiratory disease complex (PRDC), a multi-etiologic respiratory infection that is a major economic and animal health threat to the global swine industry [1]. Uncomplicated IAV infections are estimated to cost $3.23 per head placed, while more complicated infections can cost over $10 per head [2]. IAV is highly contagious in pigs and multiple subtypes circulate endemically in the domestic swine population: H1N1, H1N2, and H3N2 [3]. Though H1N1 isolates are the most common IAV isolated from swine, H3N2 isolates circulate within herds and novel isolates are frequently introduced from humans in spillover events [3].

IAV is most commonly transmitted between pigs through direct contact with oronasal secretions from infected animals [4, 5], where it infects the epithelial cells of the respiratory tract. Replication of IAV occurs predominantly in the lower respiratory tract; however, nasal replication also occurs and can contribute to changes in the nasal epithelial mucosa [5]. The nasal mucosa serves as an important physical barrier to infection and harbors the native microbiota, which has a protective role against infection by aerosol-transmitted pathogens [6, 7]. The nasal microbiota also hosts a number of potential pathogens or pathobionts, including *Bordetella bronchiseptica*, *Glaesserella parasuis*, *Streptococcus suis*, *Actinobacillus pleuropneumoniae*, *Actinobacillus suis*, and *Trueperella pyogenes*—all of which play a major role in PRDC [1, 8]. In humans, stressful conditions and alterations in the epithelial barrier, such as viral infections, can perturb the microbiota and allow pathobionts to overgrow and contribute to complex respiratory infections [7, 9].

The microbiota within the nasal cavity can be easily and repeatedly sampled, which makes longitudinal studies feasible and enables examination of the impact of viral respiratory infections, such as IAV, on the swine nasal microbiota. Two studies have investigated the effect of IAV infection on the swine nasal microbiota. One evaluated the acute phase of IAV infection (through day 5 post-challenge), while the other investigated through the resolution of IAV infection to day 31 post-challenge [10, 11]. Both studies found reduced microbial richness post-challenge with IAV, but no change in the time-dependent development of the microbiota [10, 11]. However, small group sizes and inter-animal variation may have impacted the results of these studies. Additionally, the reports evaluating the respiratory microbiota in humans following IAV infection have showed varied results. Studies have found the nasal microbiota of humans to have no changes, subtle transient changes, or significant disruption associated with IAV infection [12–16]. Changes in the human nasal microbiome after infection with IAV have included changes in the abundance of dominant microbial populations and changes in microbial diversity [14–16], which has been associated with the severity of influenza symptoms [17].

Though the impact of IAV infection on the swine nasal microbiota has not been thoroughly investigated, examination of IAV co-infection with important respiratory pathobionts in swine, such as *G. parasuis*, *A. pleuropneumoniae*, and *B. bronchiseptica*, has detected significant impairment of the host immune responses [18] and increased severity of disease [19–22]. In humans, the alteration in host immune function and damage to the nasal mucosa associated with IAV infection has been shown to disturb the nasal microbiota by reducing anaerobes associated with the healthy core microbiota and increase the abundance of respiratory pathogens present in the nasal cavity [16]. The impact of these disturbances has the potential to predispose the host to more complicated, secondary respiratory infections. To further define the impacts of IAV on the nasal microbiota of swine and better understand how changes to the microbiota may impact the development of secondary bacterial infections, we examined the longitudinal effects of IAV exposure by characterizing the nasal microbiota of pigs inoculated with IAV or sham inoculated through 16S rRNA sequencing of the hypervariable V4 region over a 42-day period.

## Materials and methods

### Animal study design and sample collection

A total of 20 weaned, three-week-old, crossbred pigs were obtained from an IAV negative herd. Pigs were randomly distributed in two ABSL2 rooms with 10 pigs in each room. Pigs were clinically healthy upon arrival and acclimated for 12-days prior to the start of study. Animals were transitioned to NexGen 14 Complete ration for finishing pigs (Kent Nutrition Group, Inc, Muscatine, IA) prior to study start. Guaranteed analysis can be found in Additional file 1. Pigs were not treated with antibiotics before or during the study. On the day of challenge (day 0), nasal samples were collected from pigs as described below, followed by intranasal inoculation with 2 mL (1 mL per nostril) of 10^6^ TCID_50_/mL H3N2 swine influenza virus isolate A/Swine/Missouri/A01840724/2015 [23] for the IAV group and 2 mL (1 mL per nostril) of PBS for the control group. The IAV strain A/Swine/Missouri/A01840724/2015 was selected as a representative isolate of the 2010.1 HA clade, which is the most frequently detected H3 clade in U.S. swine [23]. Pigs were evaluated daily for presence of clinical signs of respiratory disease, including coughing, sneezing, nasal discharge, lethargy, and inappetence. Animals were sampled on the following days after challenge: 1, 3, 7, 10, 14, 21, 36, and 42. On day 42, animals were humanely euthanized and necropsied.

Samples of the nasal microbiota were obtained via nasal wash followed by nasal swab. Sterile 1xPBS (5 mL) was applied via syringe into the nostrils and effluent PBS was collected. FLOQSwabs (Copan Flock Technologies, Murrieta, CA, USA) were inserted into each nostril and placed in the collected nasal wash. Nasal samples were vortexed and the nasal swab was removed prior to centrifugation at 10 000 rpm for 10 min at 4 °C. The supernatant was decanted and the pellet was resuspended in 200 µL of PBS. Samples were then applied to a 96-well plate and stored at −80 °C for subsequent DNA extraction.

### Viral propagation and assessment of viral infection

IAV was propagated in Madin-Darby canine kidney (MDCK) cells and clarified as previously described [24]. Titers were performed on clarified inoculum and inoculum was frozen at − 80 °C until use. Viral titration in MDCK cells was performed as described previously [24] and analyzed using the Reed and Muench method [25]. Following inoculation, viral infection was verified using the IDEXX ELISA evaluating total serum antibody to influenza nucleoprotein (IDEXX Laboratories, Westbrook, ME). Serum samples from day 0 and day 42 were diluted 1:10 and the ELISA was run and assessed according to the manufacturer’s recommendations reading at a wavelength of 650 nm.

### DNA extraction, amplification, and Illumina sequencing

Bacterial DNA was extracted from samples with the PowerMag Microbiome DNA/RNA Isolation Kit (Qiagen, Hilden, Germany) and the epMotion workstation (Eppendorf, Hamburg, Germany) following manufacturer’s instructions. DNA concentration was assessed with the Quant-IT PicoGreen dsDNA Kit (Thermo Fisher Scientific, Waltham, MA, USA). All samples were processed through the MiSeq Wet Lab SOP to prepare the hypervariable V4 region of 16S rRNA gene sequences for Illumina MiSeq sequencing [26, 27]. Mock community and negative extraction controls were also included for sequencing [28]. Once samples were processed, pooled, and normalized to at least 1 nM, they were submitted to the NADC Genomics Facility in Ames, IA for preparation of 250 bp paired-end library and sequencing on the MiSeq instrument (Illumina, Inc. San Diego, CA, USA) using version 2 chemistry.

### Sequence processing and taxonomy assignment

Raw fastq data were retrieved from the MiSeq platform and processed using mothur (version 1.48.0) and MiSeq SOP [26, 29]. Sequences were assigned taxonomies with the SILVA release 132 database [30, 31] and identified a total of 1741 operational taxonomic units (OTUs). After processing sequences and subsampling all samples to 4277 sequences with mothur, which resulted in a total of 1393 OTUs, seven samples for the IAV group and ten samples for the control group were analyzed for every day of the study. Raw sequence data were deposited as FASTQ files in the Sequence Read Archive of the National Center for Biotechnology Information (SRA NCBI) under Bioproject PRJNA525911.

### Nasal microbiota data and statistical analyses

Data analysis and figure generation were conducted using R version 4.1.2. The R packages ggplot2 [32], scales [33], cowplot [34], and plotly [35] were used for figure generation. The R packages vegan [36], phyloseq [37], philentropy [38], dplyr [39], and tidyverse [40] were used to analyze various alpha (Shannon, Inverse Simpson) and beta (Bray–Curtis dissimilarity matrices based on OTU abundances) diversity measures of the samples from each treatment group (IAV, control) and day. To assess for any significant differences in alpha and beta diversity between treatment groups on a given day (*p* ≤ 0.05), Wilcoxon rank sum test and Permutational Multivariate Analysis of Variance using Distance Matrices (adonis) function [41] were conducted on the subsampled data, respectively. The average number of distinct OTUs per day for each treatment was calculated, and a two-way ANOVA was performed in GraphPad Prism 9 (Dotmatics, San Diego, CA, USA).

Raw data (non-subsampled data with singletons removed) was filtered to remove samples that had fewer than 4,277 sequences and OTUs that occurred less than 10 times globally. To identify differentially abundant OTUs between groups, DESeq2 package version 1.34.0 [42–44] was used. OTUs were combined at the genus-level and the Benjamini–Hochberg adjustment was used to limit the false discovery rate of contrasts. The significance of the log_2_ fold change of comparisons was determined using the Wald test and a cutoff of *p* ≤ 0.05. OTUs that were differentially abundant (log_2_-fold changes with adjusted *p*-values < 0.05), and showed no significant differences between the two groups on day 0 were selected for further study. Genera associated with porcine respiratory disease complex were also examined even if they were significantly different on day 0 to observe if changes in relative abundance occurred.

## Results

### IAV clinical presentation and antibody titers

Following challenge, animals were monitored twice daily for clinical signs of IAV infection. Pigs remained clinically normal after challenge. No coughing, sneezing, or nasal discharge were observed in IAV challenged animals. One animal in the IAV group developed neurologic signs on day 18 and was euthanized. This animal was excluded from the analysis. Attempts to quantify nasal IAV shedding were unsuccessful. To confirm IAV infection, IDEXX ELISA was used to evaluate serum antibody levels to the nucleoprotein on day 0 and day 42 (Additional file 2). Antibody levels on day 0 were negative for all animals in both groups. On day 42, all animals in the control group were negative for nucleoprotein antibody. Most of the IAV group (7/9) seroconverted during the study and were positive on day 42. The seven animals that seroconverted to IAV and were alive throughout the study period were included in the microbiome analysis.

### Community and diversity changes in the nasal microbiota as a result of IAV infection

**Figure 1:**
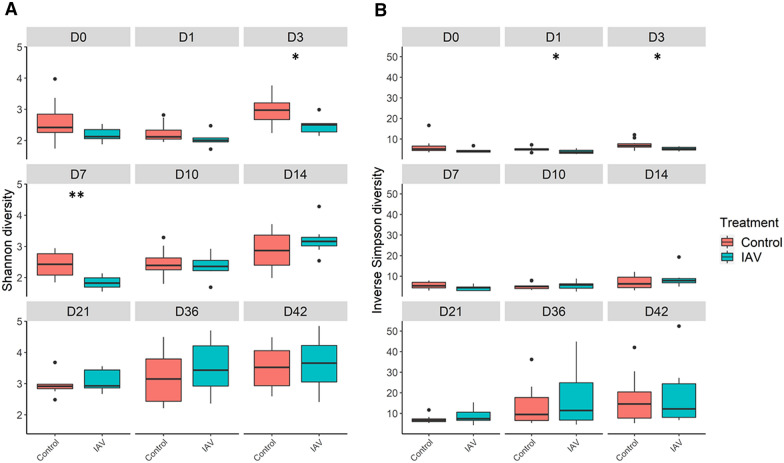
**Alpha diversity comparisons between IAV and control groups.** IAV had significantly different Shannon diversity on days 3 (*) and 7 (**) relative to control (A), and significant differences in Inverse Simpson diversity relative to control were observed on days 1 (*) and 3 (*) (B). Each panel, from left to right, top to bottom refer to day 0 through day 42. The groups are indicated in the legend on the right: IAV (influenza A virus) = blue, and control = red. Box and whisker plots indicate the interquartile range (IQR) and 1.5 times the IQR. Outliers (higher or lower than 1.5 times the IQR) are indicated by black circles. Pairwise comparisons within groups across time and between groups at each time point were performed using the Wilcoxon rank sum test at a significance level of 0.05 (*p* < 0.05:*; *p* < 0.005:**).

Community richness was compared between the control and IAV groups. Significant differences in community richness were not observed at the majority of time points post-challenge (Figure 1). However, there were statistically significant differences early in the study. There was a significant difference in the Shannon diversity index between IAV and control groups on days 3 and 7, with IAV having a lower diversity than control (*p* < 0.05) (Table 1). The Inverse Simpson diversity index indicated significant changes in diversity between the two groups on days 1 and 3 (Table 1). Using either index, IAV infection had minimal effect on the nasal microbial community richness following day 7. When the average number of distinct OTUs per day for each treatment group was calculated, no statistical significance was observed between the IAV and control groups. However, there were significantly increased OTUs in both groups on day 36 and 42 compared to earlier days of the study (Additional files 3 and 4).

**Table 1:** **Pairwise Wilcoxon rank sum test between the IAV and control groups on each day for the alpha diversity values (Shannon and Inverse Simpson) (*p*** < 0.05:*; *p* < 0.005:**).

Day	Shannon	Inverse Simpson
0	0.193	0.070
1	0.161	0.043*
3	0.033*	0.033*
7	0.005**	0.088
10	0.740	0.601
14	0.315	0.601
21	0.669	0.315
36	0.364	0.669
42	0.813	0.887

**Figure 2:**
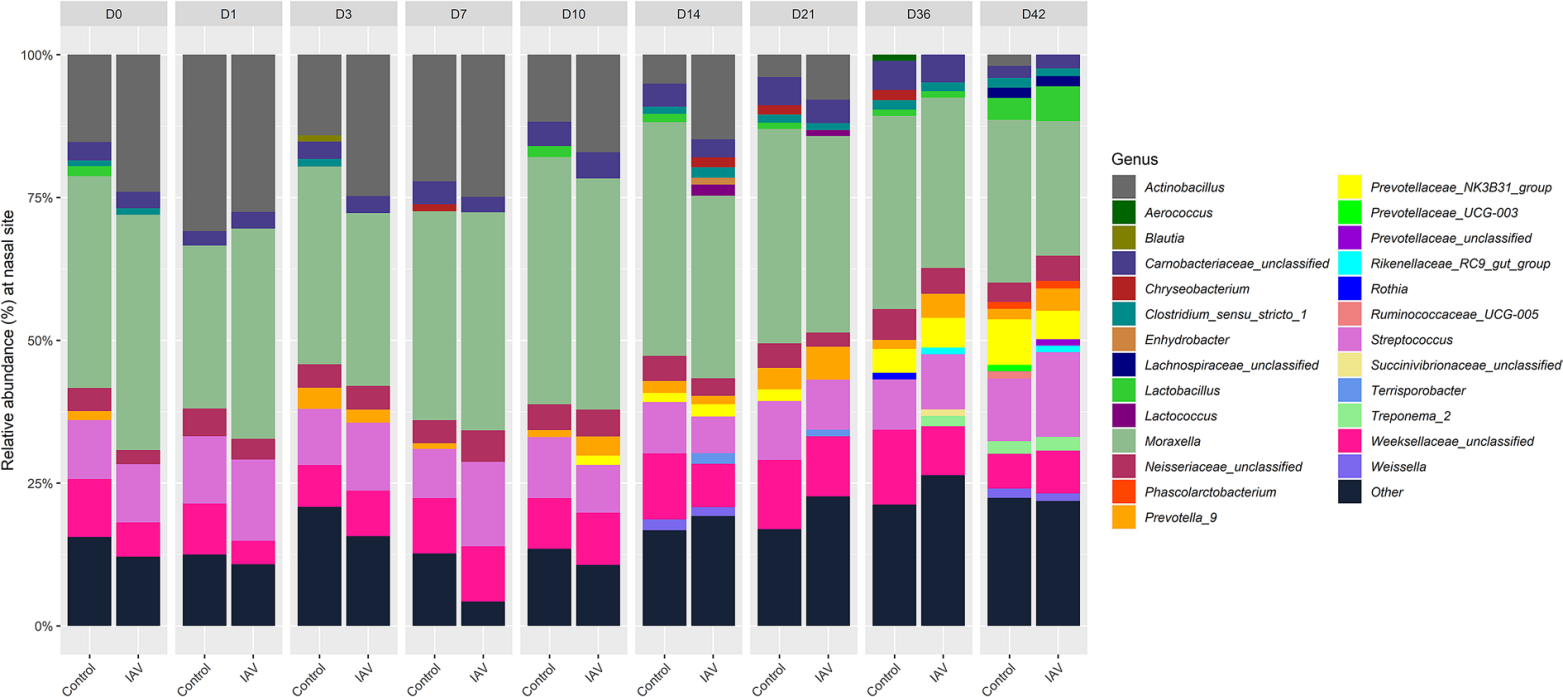
**Relative abundances of nasal bacterial genera over the 42-day period between IAV and control groups.** Within each day (day numbers listed at the top of the bar plots), each bar plot shows the average percentage of total genera found in each group. Only genera with more than 1% abundance are shown. Genera with less than 1% abundance are grouped in the “Other” category. *IAV*  influenza A virus.

To visualize and assess the changes in the nasal microbial community composition between the IAV and control groups on a given day, non-metric multidimensional scaling (NMDS) ordination (Additional file 5) and a series of pairwise PERMANOVA tests (Table 2) assessing rarefied OTU abundance were performed comparing the IAV and control groups at each day. From the day of challenge (day 0) through day 10, no significant differences in the nasal microbial community compositions between control and IAV groups were observed. However, on day 14 and 21, significant differences in the community composition were detected (*p* < 0.05, 0.018 < *R*^*2*^ < 0.027). The nasal microbial communities are visualized in Figure 2, which depicts the relative abundance of genera present in the nasal cavity throughout the study.

**Table 2:** PERMANOVA tests of associations of nasal bacterial community structure between control group and influenza A (IAV) group on a given day (*p* < 0.05:*).

Day	F Model	R^2^	*p*-value
0	1.702	0.102	0.165
1	1.708	0.102	0.165
3	1.772	0.106	0.165
7	1.623	0.098	0.165
10	1.440	0.088	0.198
14	4.395	0.227	0.018*
21	2.965	0.165	0.027*
36	2.613	0.148	0.144
42	1.903	0.113	0.165

**Figure 3:**
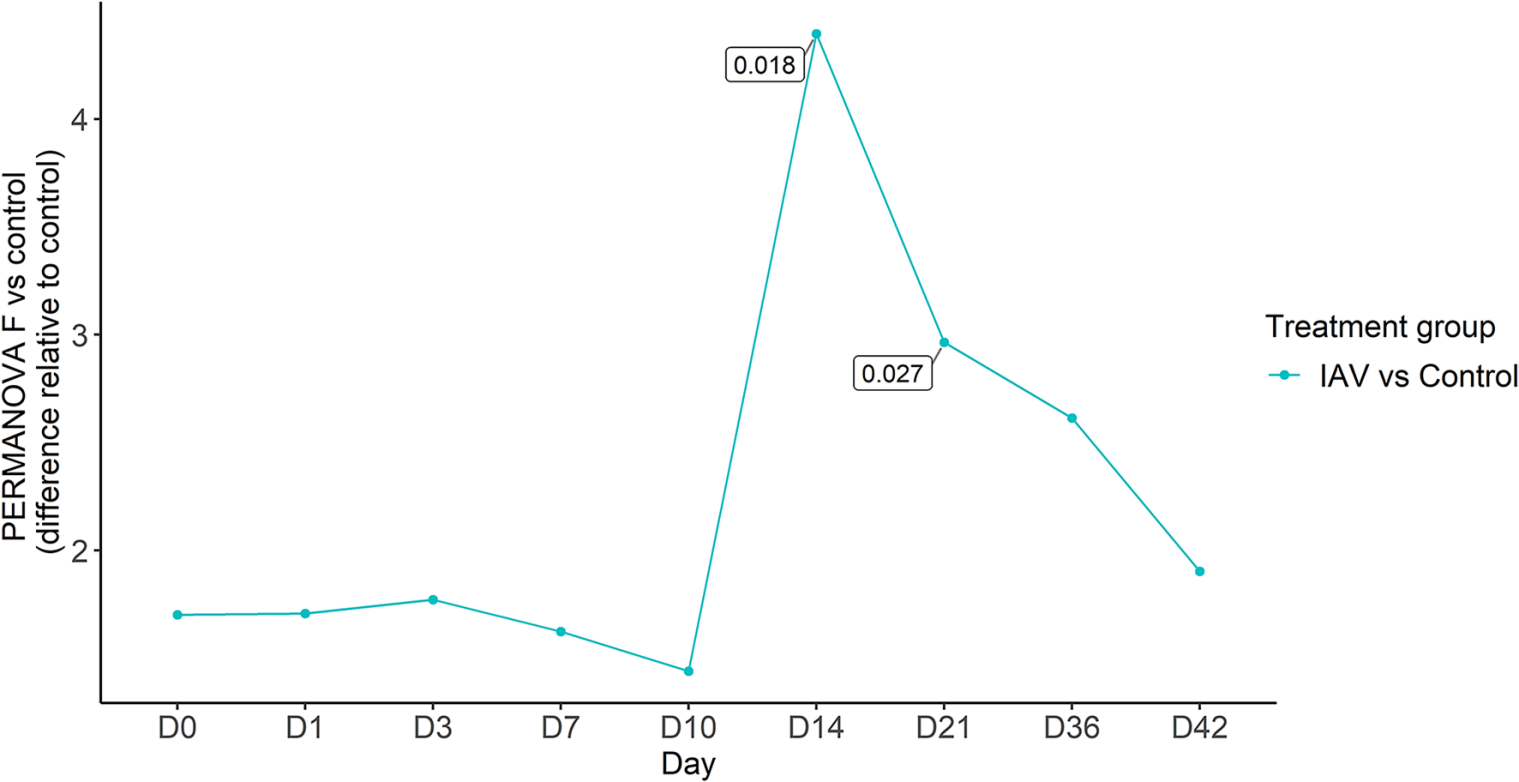
**The community structure of the nasal microbiome of the IAV group significantly differed from that of the control group on days 14 and 21.** The *F*-statistics calculated from PERMANOVA pairwise comparison of the control group versus IAV group were plotted against time (days 0 to 42). Only where bacterial community structure of the IAV group is significantly different from control group are *p*-values shown. *IAV* (influenza A virus)  blue.

To visualize the magnitude of change in the community of the nasal microbiome between the IAV and control groups over time, *F-*statistics from PERMANOVA tests comparing the IAV group to the control group were plotted at each sampling day (Figure 3). After challenge, no significant changes were observed from days 1 through 10 for the IAV group when compared to the control group. However, by day 14, the *F*-statistic (4.40) was at its highest, with the days following (days 21 through 42) ranging between 1.90 and 2.96. Though the difference between the IAV and control groups peaked on day 14, changes occurring on day 21 were significant and differentiated the nasal microbiota of the IAV group from the control group (Figure 3).

The variables treatment, day, and treatment-by-day were examined with the Adonis function to determine the level each variable contributed to the observed shifts in the nasal community compositions between IAV and control groups. The treatment, (*p* = 0.0009) day, (*p* = 0.0001) and treatment-by-day (*p* = 0.0006) variables exhibited significantly strong effects (Table 3). The day of sampling was the strongest contributor to the variation observed in the nasal microbial community between the two groups, followed by treatment-by-day, and lastly treatment (Table 3). Therefore, time, not treatment, had the largest overall impact on changes to the nasal microbial community.

**Table 3:** **Adonis test measuring impact of day, treatment, and treatment-by-day.**

Variable	R^2^	*p*-value
Day	0.375	0.0001
Treatment	0.017	0.0009
Treatment-by-day	0.063	0.0006

### Differentially abundant OTUs in the nasal cavity in response to IAV challenge

**Figure 4:**
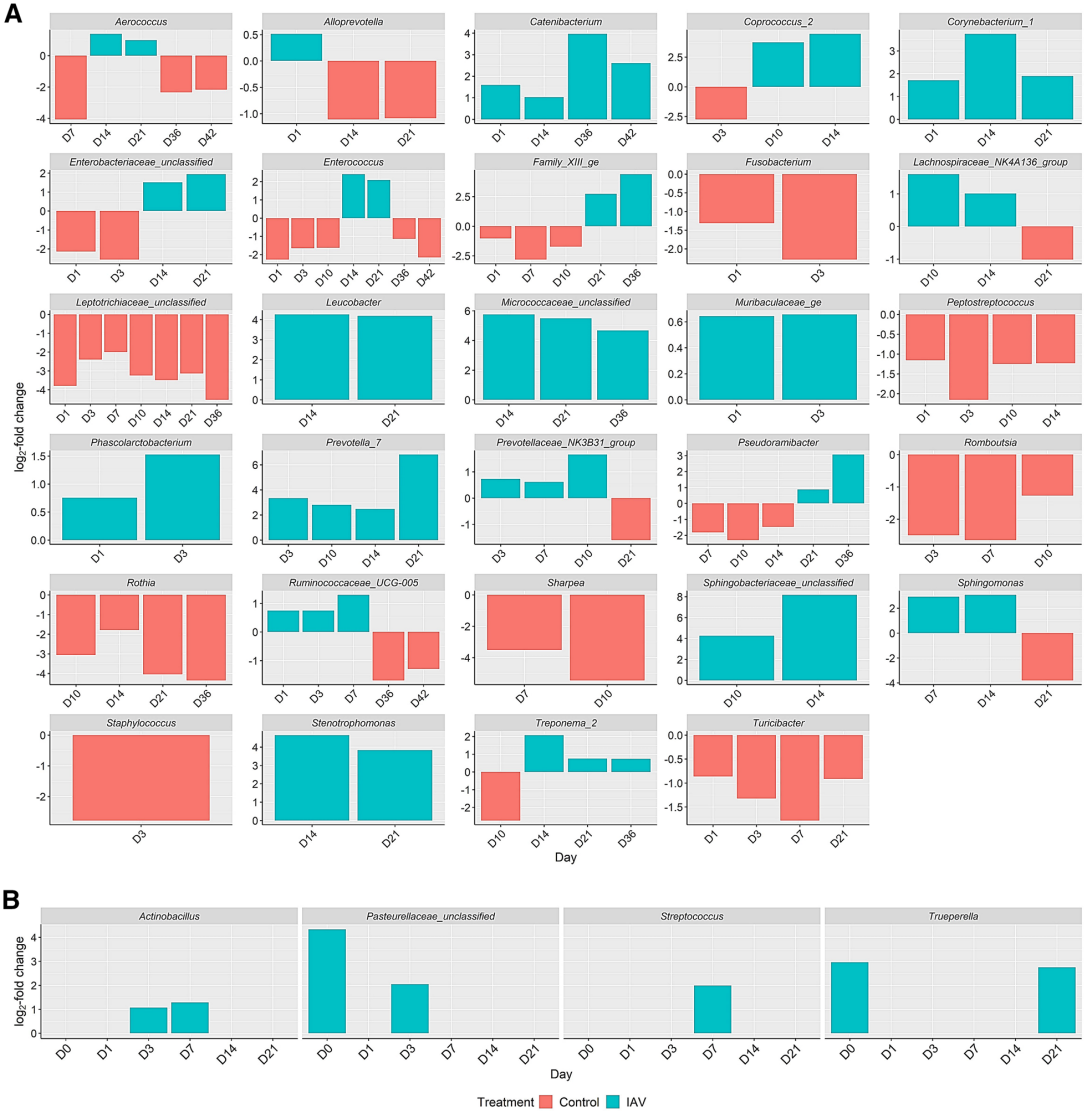
**An abbreviated list of nasal bacterial genera with significant differential abundances between IAV and control groups.** Differentially abundant genera between groups were determined using the DESeq2 package. The x-axis represents the day (D3 = day 3, D7 = day 7, etc.), y-axis displays the log_2_-fold changes of the genera, and corresponding genus is listed above each panel. Control = red, IAV (influenza A virus) = blue. Genera of interest different than those statistically different day 0 are shown in 4A. Genera associated with PRDC are shown in 4B, even if statistically significant abundances were present on day 0.

To determine differentially abundant OTUs between the two groups over the 42-day period, we used the DESeq2 R package. All OTUs that showed significant log_2_-fold changes were identified and are listed in Additional file 6. There were 137 genera with comparable abundances on day 0 that showed significant differences in abundance between the groups following IAV challenge. Genera of note that were differentially abundant between the IAV and control group, which showed trends during acute infection (days 1–7) or contributing to the perturbance between IAV and control groups on days 14 and 21 are shown in Figure 4A.

The greatest differences between IAV infected animals and control animals were seen after IAV resolution on days 14 and 21. In the IAV group, increased abundances of *Aerococcus*, *Cornebacterium 1*, *Enterobacteriaceae* (unclassified), *Enterococcus*, *Leucobacter*, *Micrococcaceae* (unclassified), *Prevotella 7*, *Stenotrophomonas*, and *Treponema 2* were observed on both days 14 and 21 (Figure 4A). Several of these genera are facultative anaerobic or anaerobic bacteria commonly found in the gastrointestinal tract, including *Enterobacteriaceae*, *Enterococcus*, and *Prevotella 7*. In the control group, increased abundance of *Alloprevotella*, *Leptotrichiaceae* (unclassified), and *Rothia* were observed (Figure 4A). The genus *Alloprevotella* has been found in the gastrointestinal tract of swine [45]. *Rothia* has been associated with oral mucous membranes, and *Leptotrichiaceae* has been associated with the tonsillar microbiome of newborn piglets [46].

To assess the impact of IAV infection on the abundance of respiratory pathogens involved in PRDC, OTUs of respiratory significance were evaluated, including *Actinobacillus*, *Pasteurellaceae* (unclassified), *Streptococcus,* and *Trueperella* (Figure 4B). *Actinobacillus*, which may include the respiratory pathogens *Actinobacillus suis* and *Actinobacillus pleuropneumoniae*, showed significantly higher abundance in the IAV group on days 3 and 7 post-challenge. *Pasteurellaceae* (unclassified), which may include the species *Glaesserella parasuis* and *Pasteuruella multocida*, was found in significantly higher abundance in the IAV group on day 0, returned to comparable levels on day 1 and became elevated in the IAV group on day 3 before returning to comparable levels (Additional file 6, Figure 4B). *Streptococcus*, which may include the species *Streptococcus suis*, a PRDC pathogen, as well as other Streptococcal species, was significantly elevated on day 7 (Figure 4B). *Trueperella* was significantly higher on day 0 for the IAV group compared to the control; *Trueperella* returned to comparable levels before becoming elevated on day 21 for the IAV group compared to the control (Figure 4B). Although differences were observed among *Actinobacillus, Pasteurellaceae* (unclassified), *Streptococcus,* and *Trueperella*, there were no significant differences observed on any day between IAV and the control group for *Mycoplasma*, which may have encompassed respiratory pathogens *Mycoplasma hyopneumoniae* and *Mycoplasma hyorhinis*.

## Discussion

There are many unanswered questions about the impact of changes in the upper respiratory microbiota on PRDC infections. Initial investigations into the impact of IAV on the nasal microbiota of pigs examined animals only during the acute phase of infection or utilized small group sizes, which can reduce confidence in the analysis [10, 11]. Additionally, previous research has shown co-infections between IAV and important swine respiratory pathobionts or pathogens alter the host response to infection and worsen the severity of disease [18–22]. To better understand the impact of IAV infection on the nasal microbiota and how changes may contribute to secondary bacterial infections, this study characterized bacterial communities in the swine nasal microbiota after IAV challenge using a longitudinal approach.

In this study, we found IAV infection did not result in reduced distinct OTUs, caused no consistent changes to species richness, and resulted in no significant persistent disturbance to community composition compared with the control group during acute infection (D1-7). This contrasts with previous research that identified reduced community richness and changes to the taxonomic composition of the nasal microbiota in IAV infected pigs [10, 11]. The differences in our results could be due to differences in data analysis methods, the use of different IAV subtypes/strains, or the use of larger group sizes which reduced the impact of inter-animal variation on the analysis. Our study grouped sequences into OTUs rather than ASVs [10], which alters the resolution at which the data is analyzed. However, previous work indicates OTU data can fully represent the community richness and alpha diversity captured using ASV data [47]. Chiarello et al. also observed a high correlation between ASV and OTU analysis for alpha diversity (Shannon) and beta diversity (Bray–Curtis) measurements [47], which were used in this study, indicating the impact of using the OTU classification may be minimal. Further, the evaluation of taxonomic composition in previous work examined changes at the family level compared to the genus level used in this study [10, 11]; therefore, this study increased the resolution of the analysis compared to previous work.

Second, the IAV strain used in this study could alter the changes seen in the microbiota following challenge compared to prior work. The H3N2 strain used in this study caused subclinical infection, with no observed clinical signs. Previous work in humans has shown the impact of IAV infection on the microbiota is related to the severity of infection [17]; therefore, the reduced impact of IAV infection on the nasal microbiota seen in this study could be related to subclinical infection with the H3N2 IAV strain. Finally, this study included 10 pigs per group, though the analysis retained only 7 animals in the IAV group. Increased group size is important for data with high variability between animals, which has been observed in previous work evaluating the nasal microbiota following IAV exposure [10]. With more animals in each group, the impact of differences within the group decreases, which may also contribute to the differences seen between this study and previous research that evaluated the impact of IAV on the swine nasal microbiota.

Although the nasal microbiota in the IAV group was significantly different from the control group on days 14 and 21, the largest effect on the variation seen in the nasal microbial community composition was found to be time. This suggests IAV infection had minimal impact on the nasal microbiota and did not impact the community long term, as the communities are similar from day 36 through the end of study. This is consistent with previous research, which did not see a long-term impact on the respiratory microbiota or changes to the time-dependent development of the nasal microbial community in pigs following IAV infection [10]. The perturbations in the IAV group after IAV resolution (days 14 and 21) are likely due to the life stage of the pigs. This study was completed in pigs following weaning, and at this age, the nasal microbiota is still developing [48], which also may affect our ability to fully elucidate the impact of IAV infection versus community development on the nasal microbiota.

Though no major changes were observed in the beta diversity or overall community composition of the pigs’ nasal microbiota during IAV infection (day 1–7), there were significant differences in abundances of genera containing important respiratory pathogens between infected and non-infected animals. We observed increased relative abundances of several genera in the IAV group that have been associated with animal disease status. For one, we observed increased abundances of *Actinobacillus* and *Streptococcus* in nasal microbiota of IAV group compared to control group during acute IAV infection. These two genera would include important PRDC bacterial pathogens, though this study did not identify organisms to the species level. In addition, increased abundance of *Actinobacillus, Neisseriaceae* (unclassified), and *Streptococcus* were seen in the IAV group on day 7 post-challenge (Figure 4B, Additional file 4). This change has been seen previously in the nasal microbiota of pigs from farms with a history of Glӓsser’s disease outbreaks when compared with pigs from farms with no history of Glӓsser’s disease and in diseased animals when compared to healthy animals on the same farm [49]. Additionally, our work observed similar changes to previous work [10, 11], with increases in the *Pasteurellaceae* and *Neisseriaceae* families following IAV infection (data not shown). The shifts in abundance of pathogen containing genera seen in this study raise questions about the development of secondary bacterial infection and prompt further studies to assess the consequences of changes in the abundance of these genera and how these changes can impact the respiratory health of swine. However, the taxonomic assignment in this study was limited to the genus level, which restricts the assessment of individual members of the population at the species level.

In conclusion, IAV infection had no major effect on swine nasal microbial diversity or microbial community composition. Following IAV infection resolution (days 14 and 21), there was a significant change in the composition of the nasal microbiota of the IAV group compared to control group; however, these changes were temporary, and the communities were similar on days 36 and 42. There were also significant changes observed during the study period in the abundance of: presumed commensal organisms, genera previously associated with the development of Glässer’s disease, and nasal genera which may include PRDC pathogens. The significance of the changes in abundance of these organisms is unknown at this time, which highlights the need to further investigate the consequences that perturbation of the nasal microbiota can have on the protective role of the microbiota, host immune response, and overall respiratory health of the pig.

## Supplementary Information

**Additional file 1. Guaranteed analysis of finisher feed**. Feed components as a percent of the total ration, parts per million (ppm), or international units per pound (IU/lb) within the finisher ration. Components are presented as a minimum, maximum, or range contained within the feed.

**Additional file 2.** **Sample-to-negative (S/N) ratio for IDEXX Swine Influenza Virus Antibody Test (ELISA)**. Animals are considered positive when the S/N ratio is < 0.6. An asterisk (*) denotes a positive result. Most of the animals in the IAV group seroconverted (8/10), while none of the control animals seroconverted (0/10).

**Additional file 3.** **Mean number of distinct OTUs**. The mean distinct OTUs for each group on each sampling day is represented. No statistical differences between IAV and control groups were found at any timepoint.

**Additional file 4.** **Statistical output of two-way ANOVA comparing the average number of distinct OTUs for the IAV and control groups**. The mean distinct OTUs increased over time in both groups, but no statistical differences were noted between groups at any time point.

**Additional file 5.** **NMDS plot of the nasal microbial communities**. Non-metric multidimensional scaling (NMDS) ordination is another way of visualizing the data underlying the PERMANOVA results and was generated using the Bray–Curtis dissimilarity metric calculated with rarefied OTU abundance data (k = 2, stress = 0.165). The plot is split into 9 treatment-by-day panels. The labels at the top of each panel refer to the day sampled (D0 = day 0, D1 = day 1, etc.) Day 0 samples were collected before the animals in IAV group were challenged. Each point represents one sample. The closer samples are to each other the more similar the microbial compositions of the samples are. Samples are linked to the treatment group centroid by segments and the standard error of the treatment group is depicted with an ellipse. Within each day are the two groups: control = red, IAV (influenza A virus) = green. Significant differences in the nasal microbial composition between control and IAV group were observed on days 14 and 21.

**Additional file 6.** **Differentially abundant operational taxonomic units (OTUs) based on PERMANOVA pairwise comparisons**. A list containing individual OTUs with differential abundance between the IAV and control groups at different time points. Each OTU listed has relative log-fold change, absolute abundance, and taxonomic classification.

## Data Availability

Raw sequence data were deposited as FASTQ files in the Sequence Read Archive of the National Center for Biotechnology Information (SRA NCBI) under Bioproject PRJNA525911. The R code and input files used for data analysis are available on github [50].
