# Supplementary material for: Resilience of swine nasal microbiota to influenza A virus challenge in a longitudinal study
Source: Vet Res. 2023 May 2;54:38. doi: 10.1186/s13567-023-01167-9 (PMC10152739; doi:10.1186/s13567-023-01167-9)
Supplement: Supplementary file 1 — Additional file 1. Guaranteed analysis of finisher feed. Feed components as a percent of the total ration, parts per million (ppm), or international units per pound (IU/lb) within the finisher ration. Components are presented as a minimum, maximum, or range contained within the feed. [file 13567_2023_1167_MOESM1_ESM.docx]

| **Component** | **Amount** |
| --- | --- |
| Crude protein | 14.0 % (minimum) |
| Lysine | 0.7 % (minimum) |
| Crude fat | 3.0% (minimum) |
| Crude fiber | 5.0% (maximum) |
| Calcium | 0.4-0.9% |
| Phosphorus | 0.45% (minimum) |
| Salt (NaCl) | 0.2-0.7% |
| Selenium | 0.3 ppm (minimum) |
| Zinc | 90 ppm (minimum) |
| Vitamin A | 1700 IU/lb (minimum) |
| Vitamin D3 | 435 IU/lb (minimum) |
| Vitamin E | 8 IU/lb (minimum) |

**Additional file 1 Guaranteed analysis of finisher feed.**
