# Supplementary material for: Resilience of swine nasal microbiota to influenza A virus challenge in a longitudinal study
Source: Vet Res. 2023 May 2;54:38. doi: 10.1186/s13567-023-01167-9 (PMC10152739; doi:10.1186/s13567-023-01167-9)
Supplement: Supplementary file 2 — Additional file 2. Sample-to-negative (S/N) ratio for IDEXX Swine Influenza Virus Antibody Test (ELISA). Animals are considered positive when the S/N ratio is < 0.6. An asterisk (*) denotes a positive result. Most of the animals in the IAV group seroconverted (8/10), while none of the control animals seroconverted (0/10). [file 13567_2023_1167_MOESM2_ESM.docx]

| **Animal** | **Group** | **D0 S/N** | **D42 S/N** |
| --- | --- | --- | --- |
| 152 | IAV | 0.9955 | 0.4602* |
| 153 | IAV | 0.9614 | 0.4962* |
| 154 | IAV | 1.0007 | 0.4432* |
| 155 | IAV | 0.9417 | 0.5097* |
| 156 | IAV | 0.8706 | 0.2908* |
| 157 | IAV | 0.8174 | 0.3964* |
| 158 | IAV | 0.9818 | 0.8566 |
| 159 | IAV | 0.9170 | 0.4813* |
| 160 | IAV | 0.8987 | 0.3760* |
| 161 | IAV | 0.8845 | 0.6363 |
| 162 | Control | 0.9554 | 1.0447 |
| 163 | Control | 0.9310 | 0.8467 |
| 164 | Control | 0.9471 | 0.9709 |
| 165 | Control | 0.9458 | 0.9445 |
| 166 | Control | 0.8622 | 0.9164 |
| 167 | Control | 0.8865 | 0.7132 |
| 168 | Control | 0.9835 | 0.9588 |
| 169 | Control | 0.8509 | 0.9945 |
| 170 | Control | 0.9155 | 0.9973 |
| 171 | Control | 0.8488 | 0.8953 |

**Additional file 2** **Sample-to-negative (S/N) ratio for IDEXX Swine Influenza Virus Antibody Test (ELISA).** Animals are considered positive when the S/N ratio is <0.6. An asterisk (*) denotes a positive result. Most of the animals in the IAV group seroconverted (8/10), while none of the control animals seroconverted (0/10).
