# Supplementary figures and images for: Resilience of swine nasal microbiota to influenza A virus challenge in a longitudinal study
Source: Vet Res. 2023 May 2;54:38. doi: 10.1186/s13567-023-01167-9 (PMC10152739; doi:10.1186/s13567-023-01167-9)

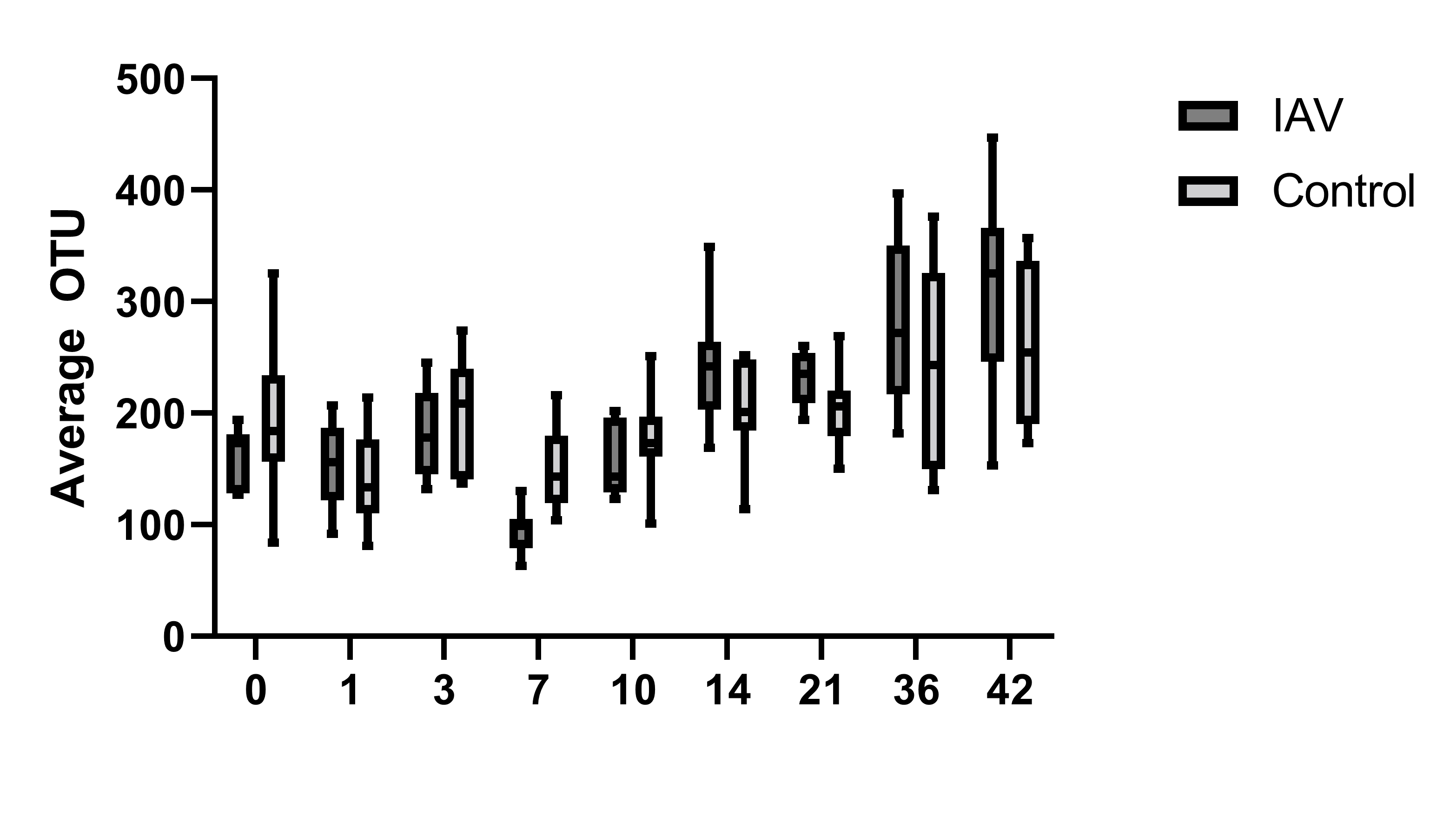

Supplement: Supplementary file 3 — Additional file 3. Mean number of distinct OTUs. The mean distinct OTUs for each group on each sampling day is represented. No statistical differences between IAV and control groups were found at any timepoint. [file 13567_2023_1167_MOESM3_ESM.tif]

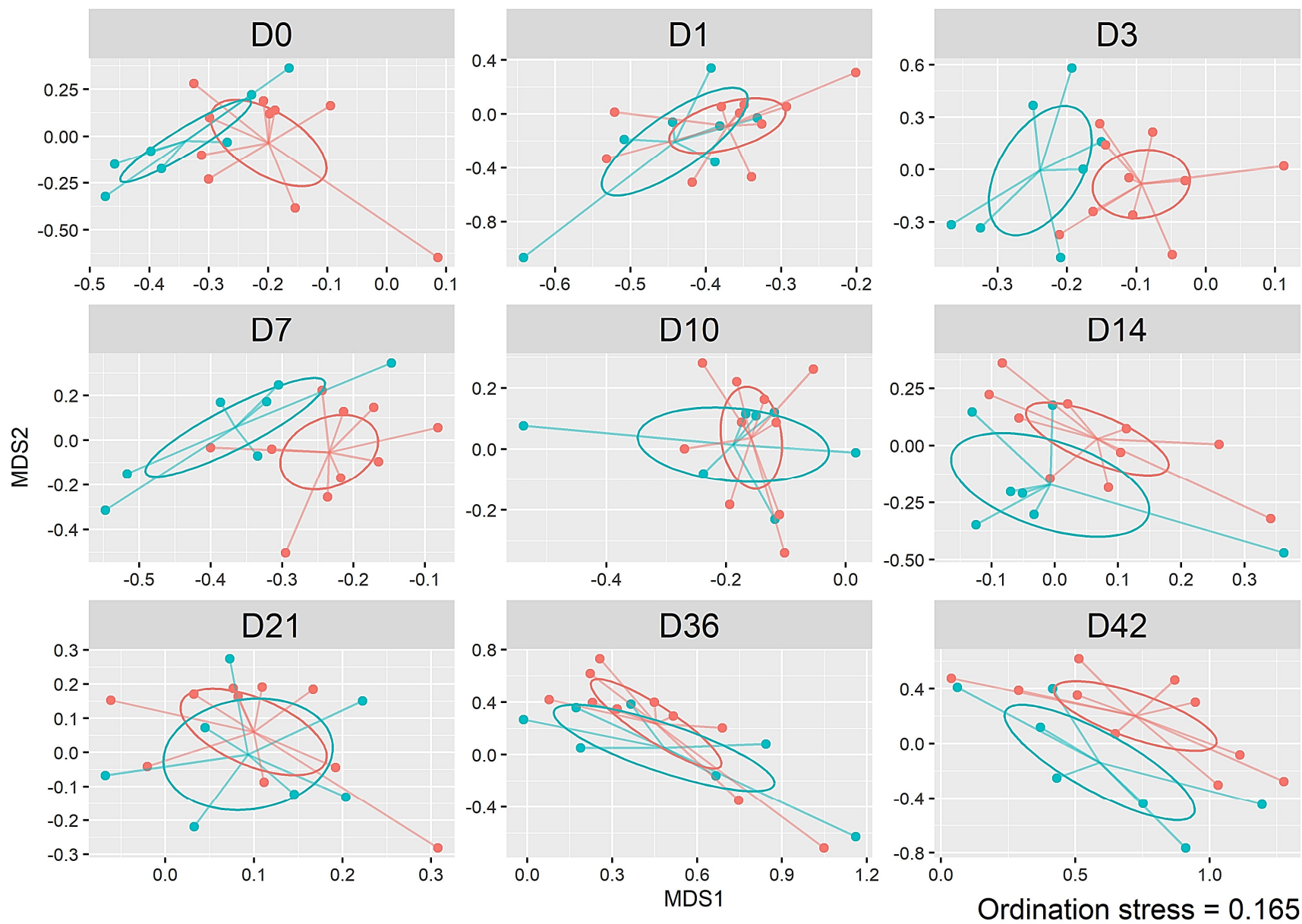

Supplement: Supplementary file 5 — Additional file 5. NMDS plot of the nasal microbial communities. Non-metric multidimensional scaling (NMDS) ordination is another way of visualizing the data underlying the PERMANOVA results and was generated using the Bray–Curtis dissimilarity metric calculated with rarefied OTU abundance data (k = 2, stress = 0.165). The plot is split into 9 treatment-by-day panels. The labels at the top of each panel refer to the day sampled (D0 = day 0, D1 = day 1, etc.) Day 0 samples were collected before the animals in IAV group were challenged. Each point represents one sample. The closer samples are to each other the more similar the microbial compositions of the samples are. Samples are linked to the treatment group centroid by segments and the standard error of the treatment group is depicted with an ellipse. Within each day are the two groups: control = red, IAV (influenza A virus) = green. Significant differences in the nasal microbial composition between control and IAV group were observed on days 14 and 21. [file 13567_2023_1167_MOESM5_ESM.pdf]
